# Modulation of Mitochondrial Dynamics in Neurodegenerative Diseases: An Insight Into Prion Diseases

**DOI:** 10.3389/fnagi.2018.00336

**Published:** 2018-11-05

**Authors:** Ting Zhu, Ji-Long Chen, Qingsen Wang, Wenhan Shao, Baomin Qi

**Affiliations:** Key Laboratory of Fujian-Taiwan Animal Pathogen Biology, College of Animal Sciences, Fujian Agriculture and Forestry University, Fuzhou, China

**Keywords:** prion diseases, neurodegenerative diseases, mitochondrial dysfunction, mitochondrial dynamics, mitophagy, therapeutic target

## Abstract

Mitochondrial dysfunction is a common and prominent feature of prion diseases and other neurodegenerative disorders. Mitochondria are dynamic organelles that constantly fuse with one another and subsequently break apart. Defective or superfluous mitochondria are usually eliminated by a form of autophagy, referred to as mitophagy, to maintain mitochondrial homeostasis. Mitochondrial dynamics are tightly regulated by processes including fusion and fission. Dysfunction of mitochondrial dynamics can lead to the accumulation of abnormal mitochondria and contribute to cellular damage. Neurons are among the cell types that consume the most energy, have a highly complex morphology, and are particularly dependent on mitochondrial functions and dynamics. In this review article, we summarize the molecular mechanisms underlying the mitochondrial dynamics and the regulation of mitophagy and discuss the dysfunction of these processes in the progression of prion diseases and other neurodegenerative disorders. We have also provided an overview of mitochondrial dynamics as a therapeutic target for neurodegenerative diseases.

## Introduction

Prion diseases comprise a group of infectious neurodegenerative disorders, including bovine spongiform encephalopathy, scrapie, chronic wasting disease, kuru, Creutzfeldt-Jakob disease, Gerstmann-Straussler-Scheinker syndrome and fatal familial insomnia. These diseases all share the same pathological characteristics of spongiform degeneration, neuronal death and astrocytic and microglial proliferation (Zhu et al., 2014; Ji et al., 2017; Shah et al., 2017a).The underlying cause of prion disease is the conformational conversion of a cellular prion protein (PrP^C^) to a misfolded isoform (PrP^Sc^), which has a higher proportion of beta-sheets in place of the normal alpha-helices in PrP^C^ and is protease-resistant (Prusiner, 1998; Wang et al., 2015). All prion diseases are associated with the accumulation and aggregation of misfolded PrP^Sc^ in the central nervous system (CNS), which leads to neuroinflammation and neurodegeneration (Prusiner, 2001; White et al., 2003; Shah et al., 2017b).

There is growing evidence that mitochondrial damage has key roles in the pathogenesis of prion diseases and other neurodegenerative disorders (Hur et al., 2002). For example, the structural and functional abnormalities of mitochondria have been described in neurons infected with a prion strain (Choi et al., 1998; Sisková et al., 2010; Faris et al., 2017). Mitochondria are highly dynamic organelles and constantly undergo fission and fusion to regulate their morphology, size and number (Chen and Chan, 2009; Wang et al., 2009a; Toyama et al., 2016). These dynamic processes are critical for modulating mitochondrial distribution, mitophagy and cell death (Detmer and Chan, 2007; Suen et al., 2008; Itoh et al., 2013). Defects in mitochondrial dynamics can lead to the accumulation of abnormal mitochondria and contribute to cellular dysfunction. Many neurodegenerative diseases are associated with alterations in mitochondrial fusion and fission, which is largely attributable to the high metabolic rate and complex morphology of neurons (Chen and Chan, 2009; Su et al., 2010; Wilson et al., 2013; Zorzano and Claret, 2014).

## Mechanisms of Mitochondrial Dynamics: Fusion and Fission

Imbalances between mitochondrial fission and fusion have been proposed to cause neurodegenerative diseases; acute readjustment of such imbalances can have beneficial effects on mitochondrial structure and function and positively influences cell survival in various disease models (Chen and Chan, 2009; Itoh et al., 2013; Sebastián et al., 2017).

**Figure 1:**
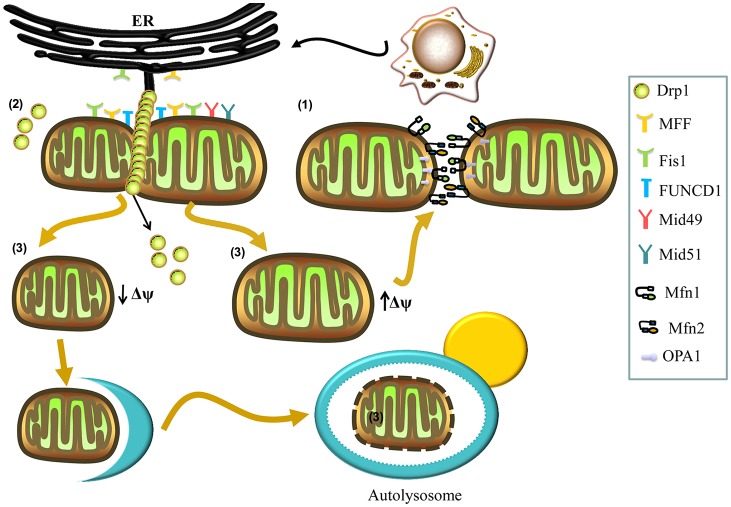
Mechanisms involved in mitochondrial fusion and fission. (1) Mitochondrial fusion is regulated by mitofusins (Mfns; Mfn1, Mfn2) and optic atrophy 1 (OPA1). The Mfn1 and Mfn2 localize to the outer membrane and form homo- and hetero-oligomeric complexes that catalyze outer membrane fusion. The OPA1 localizes to the inner membrane and interacts with Mfns to form intermembrane protein complexes that couple the fusion of the outer membrane to the inner membrane. (2) Mitochondrial fission is regulated by dynamin-related protein 1 (Drp1). In the process of fission, Drp1 is recruited to the mitochondrial outer membrane from the cytosol by various receptors (mitochondrial fission factor (Mff), mitochondrial dynamics protein 49 (Mid49), Mid51, and mitochondrial fission protein 1 (Fis1)) and oligomerizes to form spiral filaments around mitochondrial tubules, which mediate mitochondrial fission. Endoplasmic reticulum (ER) tubules also wrap around the mitochondria to mark the sites of mitochondrial division, and a population of Drp1 oligomers assembles on the ER, from where they can transfer to the mitochondria and contribute to mitochondrial fission. Subpopulations of Mff and Fis1 also localize to the ER. The FUN14 domain containing 1 (FUNDC1) is an outer mitochondrial membrane (OMM) protein that accumulates at ER–mitochondria contact sites and specifically recruits Drp1 to drive mitochondrial fission in response to hypoxic stress. (3) Finally, mitochondrial fission can generate two metabolically different types of mitochondrion, one with an increased membrane potential and a high probability of subsequent fusion, and a second type with decreased membrane potential, which is more likely to be targeted by autophagy.

Two adjacent mitochondria can fuse to form a more elongated mitochondrion through the activity of mitochondrial fusion proteins, including the conserved dynamin-related GTPases, mitofusins (Mfns) and optic atrophy 1 (OPA1; Tamura et al., 2011; Itoh et al., 2013). There are two forms of Mfn: Mfn1 and Mfn2, which localize to the outer membrane of mitochondria and form homo- and hetero-oligomeric complexes that catalyze outer membrane fusion. OPA1 localizes to the inner membrane and interacts with Mfns to form intermembrane protein complexes, which couple and fuse the outer and inner membranes (Figure 1; Cipolat et al., 2004; Song et al., 2009). Ablation of either Mfns or OPA1 in mammalian cells inhibits mitochondrial fusion, causing similar mitochondrial fragmentation phenotypes (Chen et al., 2003).

Mitochondrial fission can generate two metabolically different types of mitochondrion: one with an increased membrane potential and a high probability of subsequent fusion, and another exhibiting reduced membrane potential, which is less likely to re-fuse with the mitochondrial network and more likely to be targeted by autophagy (Figure 1; Twig et al., 2008). Mitochondrial fission is also regulated by the evolutionarily conserved dynamin-related GTPase, dynamin-related protein 1 (Drp1) in mammals and Dnm1p in yeast (Kageyama et al., 2011; Reddy et al., 2011). Drp1 is a soluble cytosolic protein, which during the process of fission, is recruited to the mitochondrial outer membrane by different receptors, including mitochondrial fission factor (Mff), mitochondrial dynamics protein 49 (Mid49), Mid51, and mitochondrial fission protein 1 (Fis1), and oligomerizes into spiral filaments that wrap spirally around the constriction points of dividing mitochondria to mediate mitochondrial fission (Smirnova et al., 2001; Figure 1). After the completion of mitochondrial fission, Drp1 spirals disassemble from the mitochondria and become available for future rounds of the process (Itoh et al., 2013; Richter et al., 2015).

Interestingly, many studies have reported contact between the mitochondria and the endoplasmic reticulum (ER), with a key role for this structure in mitochondrial fission (Friedman et al., 2011; Hoppins and Nunnari, 2012; Ji et al., 2017). ER tubules may play an active role in defining the position of mitochondrial division sites (Friedman et al., 2011), as mitochondrial division occurs at positions where the ER tethers to mitochondria (Figure 1). ER tubules wrap around the mitochondria throughout the entire fission event and mark sites where Drp1 is recruited (Friedman et al., 2011; Murley et al., 2013). Recently, Ji et al. (2017) have found that a population of Drp1 oligomers assembles on the ER, and from there they transfer to mitochondria. Furthermore, these investigators have reported that the subpopulations of Mff and Fis1 are present on the ER, demonstrating that the ER can function as a platform for Drp1 oligomerization and that ER-associated Drp1 contributes to mitochondrial division (Ji et al., 2017). Additionally, an outer mitochondrial membrane (OMM) protein, FUN14 domain containing 1 (FUNDC1), accumulates at ER–mitochondria contact sites and specifically selects Drp1 to drive mitochondrial fission in response to hypoxic stress (Figure 1). Moreover, knockdown of FUNDC1 prevents the translocation of Drp1 to mitochondria and causes mitochondrial elongation, indicating that FUNDC1 regulates mitochondrial dynamics at ER-mitochondrial contact sites under hypoxic conditions (Wu et al., 2016).

## Dysfunction of Mitochondrial Dynamics in Neurodegenerative Diseases

Mitochondria are dynamic organelles that constantly fuse with one another and subsequently break apart (in other words, undergo fission) to maintain mitochondrial homeostasis (Cagalinec et al., 2013). Fusion events serve to mix and unify mitochondrial compartments, enabling protein complementation, mitochondrial DNA repair and the equal distribution of metabolites, whereas fission acts to facilitate equal segregation of mitochondria into daughter cells during cell division and modulates the distribution of mitochondria along the cytoskeletal tracks. Additionally, fission may help to isolate the damaged segments of mitochondria, thus promoting their autophagic degradation (Twig et al., 2008; Chen and Chan, 2009). Neurons are among the most energy-consuming cell types and have a highly complex morphology; consequently, these cells are particularly dependent on mitochondrial function, including mitochondrial dynamics (Itoh et al., 2013; Rambold and Pearce, 2018). Therefore, defects in mitochondrial dynamics may lead to neuronal dysfunction. A lack of mitochondrial fusion in neurons leads to an increased mitochondrial diameter, due to swelling and aggregation of these organelles, preventing their entry into the distal, smaller diameter branches of neurites and resulting in the degeneration of mitochondria in axons and dendrites (Chen et al., 2007). Furthermore, a lack of mitochondrial fission may inhibit the isolation of damaged segments of mitochondria and their autophagic degradation, potentially promoting neuronal apoptosis (Suen et al., 2008). Dysregulation of the fusion and fission of mitochondria is associated with several neurodegenerative diseases, including prion diseases, Alzheimer’s disease (AD), Parkinson’s disease (PD) and Huntington’s disease (HD; Lin and Beal, 2006; Choi et al., 2014).

### Prion Diseases

Morphological and functional abnormalities of mitochondria are observed in CNS tissue in various prion diseases and in prion-infected experimental animals (Choi et al., 1998; Sisková et al., 2010; Park et al., 2011). Increased levels of the glutathione oxidized form and the elevated calcium content can be detected in the mitochondria of scrapie-infected mice, whereas mitochondrial membrane potential and ATP/ADP ratio are decreased in these animals (Lee et al., 2000). Prion peptides (PrP^106–126^) can perturb ER calcium homeostasis and subsequently induce the accumulation of reactive oxygen species (ROS). These processes facilitate the depolarization of the mitochondrial membrane, thus activating the mitochondria-mediated apoptotic pathway (Ferreiro et al., 2008). These findings indicate that mitochondrial dysfunction may contribute to the neurodegeneration observed in prion diseases. Additionally, several researchers have suggested that the mitochondrial fusion and fission are differentially modulated in prion diseases. For example, Choi et al. (2014) have shown that Mfn1 was upregulated in whole brains from ME7 scrapie-infected mice, and that expression levels of Fis1 and Mfn2 were elevated in the hippocampus and striatum. Furthermore, Drp1 expression was significantly reduced in the hippocampus, particularly in the cytosolic fraction, but not in the mitochondrial fraction, and the total number of mitochondria in neurons was significantly decreased, with a number of enlarged and degenerated mitochondria observed in the ME7 mouse model. These observations imply that imbalances in mitochondrial fusion and fission may contribute to the enlargement and degeneration of mitochondria that occur in the hippocampus of scrapie-infected mice. Furthermore, there is a significant decrease in Drp1 in scrapie-infected mice in the terminal stages of disease (139A, ME7 and S15), levels of OPA1 showed a tendency to decrease, and abnormalities of Drp1 and OPA1 were primarily localized to neurons. Collectively, these processes lead to the depression of mitochondrial dynamics and subsequent neuronal apoptosis (Yang et al., 2016).

Accumulating clinical and experimental evidence indicates that the excessive fission leads to mitochondrial fragmentation, with concomitant morphological changes and loss of functions (Kim et al., 2016). Li et al. (2018) have suggested that, although cellular Drp1 protein levels were decreased in prion-infected neuronal cells both *in vitro* and *in vivo*, the mitochondrial Drp1 levels increased in these prion models, thus the elevated mitochondrial Drp1 levels contributed to extensive mitochondrial fragmentation and dysfunction, as well as neuronal death and decreased synaptic plasticity (Smirnova et al., 2001).

### Alzheimer’s Disease

AD is a progressive neurodegenerative disease and one of the most common forms of dementia in adults (Peric and Annaert, 2015). Afflicted brains are characterized by the extracellular deposition of amyloid plaques composed chiefly of amyloid-beta (Aβ)-derived APP, followed by the accumulation of intraneuronal neurofibrillary tangles of hyperphosphorylated tau, which is associated with synapse and neuron loss (Hardy and Selkoe, 2002; Blennow et al., 2006). Abnormalities in the mitochondrial structure and function are reported in the brains of AD patients and associated with disease progression (Baloyannis et al., 2004). Overproduction of Aβ induces mitochondrial fragmentation and dysfunction, including increased ROS production, reduced ATP generation and lower mitochondrial membrane potential (Wang et al., 2008). Moreover, AD neurons contain a high proportion of mitochondria with broken cristae, along with significant changes in the size and number of mitochondria (Wang et al., 2009b; Bonda et al., 2010). The balance of mitochondrial fusion and fission is clearly impaired in AD neurons. For example, Wang and colleagues have observed a reduced expression in the mitochondrial fusion genes, *Mfn1*, *Mfn2* and *OPA1*, and an increased expression in the mitochondrial fission genes, *Drp1* and *Fis1*, in AD brain samples (Wang et al., 2009a). Moreover, overproduction of Aβ and the subsequent accumulation of intraneuronal tau leads to elevated Drp1 levels in mitochondria, thus mediating excessive mitochondrial fragmentation and synaptic deficiency (Wang et al., 2008; Manczak and Reddy, 2012). Furthermore, in the early stages of AD, Aβ oligomers induce significantly increased levels of mitochondrial Drp1, which interacts with Aβ monomers and oligomers to initiate the mitochondrial fragmentation (Calkins et al., 2011). In the late stages of the disease, Drp1 interacts with phosphorylated tau, which may exacerbate mitochondrial fragmentation, ultimately leading to neuronal damage and cognitive decline (Manczak et al., 2011; Manczak and Reddy, 2012).

### Parkinson’s Disease

PD is the second-most common neurodegenerative disease in humans and features the degeneration of nigrostriatal dopaminergic neurons and the accumulation of α-synuclein (Lang and Lozano, 1998; Kalia and Lang, 2015). Mitochondrial dysfunction occurs early in the pathogenesis of both sporadic and familial PD; PD cytoplasmic hybrid (cybrid) cells contain a significantly increased proportion of mitochondria with swollen vacuoles, pale matrices and few remaining cristae (Trimmer et al., 2000). Furthermore, the balance of mitochondrial fusion and fission is disrupted in several models of PD (Santos and Cardoso, 2012). An increase in phospho-Drp1 levels was observed in peripheral blood mononuclear cell samples from sporadic PD patients, highlighting an increased level of mitochondrial fragmentation. Additionally, Parkinsonism-inducing neurotoxins and 1-methyl-4-phenylpyridinium ion (MPP^+^), which are widely used to induce PD-like degeneration, trigger Drp1 translocation to the mitochondria and mitochondrial fragmentation, leading to dopaminergic cell death (Santos et al., 2015). Kamp et al. (2010) have demonstrated that α-synuclein can induce mitochondrial fragmentation by directly binding to the OMM and inhibiting mitochondrial fusion. Furthermore, these investigators found that α-synuclein does not interact directly with proteins involved in mitochondrial fusion or fission; rather it prevents lipid fusion events in proteins related to mitochondrial fusion. This led to the proposal that the influence of α-synuclein on mitochondrial dynamics is based on its interaction with membrane lipids (Kamp et al., 2010).

### Huntington’s Disease

HD is an autosomal dominant condition caused by trinucleotide expansion within a single gene, huntingtin (*HTT*) and is characterized by choreoathetotic movements and progressive emotional and cognitive disturbances (Walker, 2007; Ross and Tabrizi, 2011). There is substantial evidence from the experimental models of HD that its pathogenesis is related to mitochondrial dysfunction (Knott and Bossy-Wetzel, 2008; Chen and Chan, 2009; Su et al., 2010). Abnormalities in mitochondrial dynamics have been observed in HD brain samples, with a significant increase in the fission protein, Drp1 and decrease in the fusion protein Mfn1. Furthermore, an imbalance between the mitochondrial fusion and fission results in alterations of mitochondrial morphogenesis, which can negatively impact important cellular mechanisms and exacerbate neuronal death (Kim et al., 2010). Further, mutant HTT protein (mHTT) triggers mitochondrial fission prior to the emergence of neurological deficits and mHTT aggregates (Shirendeb et al., 2011, 2012; Reddy, 2014). Song and colleagues have found that mHTT interacts abnormally with Drp1, which, in turn, increases its enzymatic activity. Reduction of Drp1 GTPase activity can rescue mHTT-mediated mitochondrial fragmentation and defects in anterograde and retrograde mitochondrial movement and neuronal death, suggesting that Drp1 may represent a suitable target for HD therapy (Song et al., 2011).

Collectively, these findings demonstrate the important functional connection between mitochondrial dynamics (fusion and fission) and neurodegeneration in neurons. Indeed, the correct balance between the mitochondrial fusion and fission is clearly crucial for both brain development and neuronal function.

## Mitochondrial Dynamics Regulate Mitochondrial Quality Through Modulation of Mitophagy

Dysfunctional mitochondrial dynamics are a pivotal factor leading to the accumulation of defective mitochondria (Sebastián et al., 2017). A growing body of evidence from a number of neurodegenerative diseases clearly supports a major contribution from defective mitochondria to neuronal loss and the release of ROS, which are typically generated by mitochondrial respiration. ROS can cause oxidative damage to nucleic acids, lipids, carbohydrates and proteins (Trushina and McMurray, 2007; Reddy, 2010). Damaged mitochondria also release high levels of calcium and cytochrome C (cytC) into the cytosol, thereby triggering the cellular apoptosis (Suen et al., 2008). Evidently, the removal of damaged mitochondria by mitophagy (a selective form of autophagy devoted to the clearance of defective mitochondria) is essential for mitochondrial quality control and mitochondrial homeostasis (Ashrafi and Schwarz, 2013).

The mean diameter of an autophagosome is approximately 0.2–1 μm; however, the mean length of a mitochondrion, under normal conditions, ranges from several to tens of microns. Thus, before a damaged mitochondrion gets engulfed by an autophagosome, it must be fragmented and then segregated from the mitochondrial network (Mao and Klionsky, 2013; Wu et al., 2016). Hence, mitochondrial fission appears to be essential for mitophagy (Barsoum et al., 2006). Twig et al. (2008) have demonstrated that mitochondrial fission is required for the segregation of less active or damaged mitochondria from the mitochondrial network, and involves FIS1, OPA1 and Drp1; thus, a reduction of fission or an increase of fusion could inhibit mitophagy. Mitochondrial network fragmentation occurs prior to mitophagy, which is strictly coupled with mitochondrial dynamics (Rodolfo et al., 2018).

The link between mitochondrial dynamics and mitophagy is supported by experiments conducted under nutrient-deprivation conditions. Starvation-induced mitochondrial elongation protects mitochondria from autophagic turnover in mouse embryonic fibroblasts, which may permit the maximal mitochondrial ATP production crucial for the metabolic function of cells. This effect appears to be mediated by the downregulation of Drp1 by post-translational modification of two Drp1 phosphorylation sites, leading to unopposed mitochondrial fusion (Gomes et al., 2011; Rambold et al., 2011). Drp1 is also described as necessary for the mediation of various forms of mitophagy. For example, Zuo et al. (2014) have reported that during the early stage of ischemic-hypoxic stress in the rat brain, Drp1-dependent mitophagy was triggered to remove the damaged mitochondria. Inhibition of Drp1 using a pharmacological inhibitor or small interfering RNA resulted in the accumulation of damaged mitochondria, mainly through selective blocking of mitophagy. Further studies demonstrated that the decrease in mitophagy induced by inhibition of Drp1 contributed to features of apoptosis mediated by damaged mitochondria, such as ROS generation, cytC release and activation of caspase-3 (Zuo et al., 2014).

Several specific proteins contribute to the regulation of mitophagy to ensure the selective sequestration of dysfunctional mitochondria in autophagosomes and the subsequent degradation of these structures. Both parkin-dependent and parkin-independent pathways of mitophagy operate in mammals (Jin and Youle, 2013; Kageyama et al., 2015).

### The PINK1/Parkin-Dependent Mitophagy Pathway

**Figure 2:**
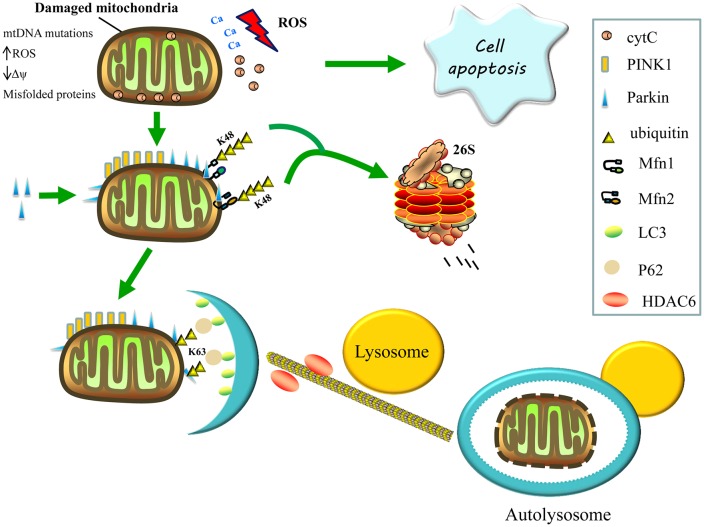
Regulation of mitophagy. Damaged mitochondria release reactive oxygen species (ROS), Ca^2+^ and cytochrome C (cytC) into the cytosol, thereby triggering apoptosis. The accumulation of PTEN-induced kinase 1 (PINK1) on the surface of damaged mitochondria then recruits parkin from the cytosol to defective mitochondria and induces the formation of K48-linked ubiquitin chains on the Mfns, Mfn1 and Mfn2, marking them for degradation by the proteasome, consequently inhibiting re-fusion of damaged and healthy mitochondria. Parkin also promotes the K63-linked polyubiquitination of mitochondrial substrates, thereby, recruiting P62 and histone deacetylase 6 (HDAC6) for the clearance of defective mitochondria. The P62 binds light chain 3 (LC3) to form autophagosomes, whereas HDAC6 activates the actin-remodeling machinery, which promotes autophagosome–lysosome fusion, thereby, enhancing the autophagy activity and degrading the damaged mitochondria.

Mammalian mitophagy is mediated by PTEN-induced kinase 1 (PINK1) and the E3 ubiquitin ligase, parkin, which are both parts of the same pathway that functions to eliminate dysfunctional mitochondria by autophagy (Eiyama and Okamoto, 2015). The loss of either protein in flies leads to mitochondrial dysfunction, and the loss of flight muscles, along with dopaminergic neurons (Park et al., 2006; Yang et al., 2006). The PINK1 is a serine/threonine kinase that contains a mitochondrial targeting sequence to facilitate its correct localization and is maintained at very low levels in healthy mitochondria by rapid proteolytic turnover. When a subset of mitochondria become damaged, PINK1 proteolysis is prevented and it accumulates on their outer membranes (Narendra et al., 2010; Youle and Narendra, 2011). The accumulation of PINK1 on the surface of damaged mitochondria induces the translocation of parkin from the cytosol to the damaged mitochondria, followed by the initiation of mitophagy (Figure 2; Pickrell and Youle, 2015).

Parkin is a cytosolic E3 ubiquitin ligase that mediates the formation of two types of polyubiquitin chains: lysine K48 linkage, which is involved in the proteasomal degradation of substrates; and K63 linkage, which is involved in autophagic degradation (Ashrafi and Schwarz, 2013). Once recruited to the mitochondria, the parkin’s E3 ubiquitin ligase activity appears to increase (Matsuda et al., 2010). Parkin may promote the K63-linked polyubiquitination of mitochondrial substrates and recruit the ubiquitin-binding autophagic components, sequestosome 1 (p62/SQSTM1) and histone deacetylase 6 (HDAC6), which are required for the clearance of damaged mitochondria. Sequestosome 1 binds the key autophagosome component, microtubule-associated protein 1A/1B light chain 3 (LC3), whereas HDAC6 activates the actin-remodeling machinery to promote autophagosome–lysosome fusion, thereby enhancing the autophagic activity (Figure 2; Lee et al., 2010; Geisler et al., 2010).

Additionally, activating signaling via the K63-linked ubiquitination of mitochondrial substrates, parkin also causes the formation of K48-linked ubiquitin chains on the mitochondrial outer surface, leading to proteasome-mediated substrate degradation (Figure 2). Parkin is also thought to ubiquitinate Mfn1 and Mfn2, marking them for proteasomal degradation, which may inhibit the re-fusion of damaged and healthy mitochondria, thereby segregating impaired mitochondria for mitophagy. These post-translational modifications of Mfn1 and Mfn2 are reduced following either PINK1 or parkin silencing (Tanaka et al., 2010; Gegg et al., 2013). Furthermore, Mfn2 appears to function upstream of parkin translocation and, aside from its role in mitophagy, downstream of parkin; Mfn2 mediates the recruitment of parkin to damaged mitochondria and subsequently binds to parkin in a PINK1-dependent manner. In the absence of Mfn2, the PINK1/parkin-dependent pathway of mitophagy is interrupted, resulting in abnormal accumulation of mitochondria in mouse cardiomyocytes and neurons; hence, Mfn2 functions as a mitochondrial receptor for parkin (Chen and Dorn, 2013).

### The PINK1/Parkin-Independent Mitophagy Pathway

Although the PINK1/parkin pathway has a dominant role in mitophagy, it is not involved in hypoxia-induced mitophagy since parkin knockdown does not prevent mitochondrial degradation by autophagy in response to hypoxia. In mammals, hypoxia-induced mitophagy is primarily mediated by FUNDC1. The FUNDC1 is a mitophagy receptor that accumulates at ER-mitochondrial contact sites by interacting with calnexin, subsequently binding to Drp1 to mediate mitochondrial fission. Ruptured mitochondria then recruit the UNC-51-like kinase 1 (ULK1) complex to initiate mitophagy and mediate mitochondrial elimination by binding to LC3 under hypoxic conditions (Wu et al., 2014, 2016). Apart from FUNDC1, other protein regulators, including Nix, cardiolipin and activating molecule in Beclin-1-regulated autophagy (AMBRA1), also contribute to the flagging and recognition of mitochondria in the PINK1/parkin independent pathway (Chu et al., 2013; Sandoval et al., 2015; Strappazzon et al., 2015).

### Impairment of Mitophagy in Neurodegenerative Diseases

Mitophagy is crucial to the elimination of damaged mitochondria and ensures the integrity and functionality of the mitochondria network. Defects in both autophagy and mitophagy have been reported as important in the onset and progression of neurodegenerative diseases (Martinez-Vicente, 2017; Rodolfo et al., 2018). In parkin-dependent mitophagy, mutations in PINK1 and parkin result in mitophagy impairment, which is related to PD (Youle and Narendra, 2011; Pickrell and Youle, 2015). Although there is an increased recruitment of parkin to damaged mitochondria in AD neurons, defective lysosomes in autophagic vacuoles are responsible for the aberrant accumulation of defective mitochondria during disease progression (Ye et al., 2015; Bordi et al., 2016). In HD, defective recognition of cargo during autophagy and the inhibition of autophagosome transport toward lysosomes result in impaired removal of damaged mitochondria, indicating that the mitophagy process is compromised (Martinez-Vicente et al., 2010; Wong and Holzbaur, 2014). Additionally, many antiapoptotic factors, such as Mcl-1 and Bcl-X_L_, along with deubiquitinases, including ubiquitin-specific protease 30 (USP30), USP15 and USP8, can antagonize the activity of parkin, thereby, inhibiting parkin-mediated mitophagy (Bingol et al., 2014; Cornelissen et al., 2014; Hollville et al., 2014; Durcan et al., 2015).

## Conclusion and Future Perspectives

Mitochondrial dysfunction is a prominent feature of several neurodegenerative diseases. Mitochondria can influence neuronal function, not only through ATP production, but also through the regulation of calcium homeostasis, synapse function, ROS generation and cell signaling and survival (Hoekstra et al., 2011). Mitochondrial dynamics (fusion/fission), along with clearance events, are important for the functional state of mitochondria. Constant fusion and fission enable the exchange between mitochondria, and the clearance of defective organelles prevents the accumulation of damaged mitochondria. Consequently, abnormalities in these processes may have deleterious effects on mitochondrial structure, function and cell survival. Based on these observations, we hypothesize that acute readjustment of dysregulated mitochondrial dynamics could be a target for protective strategies in the context of its detrimental stimulation.

Abnormal mitochondrial fission can trigger apoptosis by promoting the release of cyt C from mitochondria to the cytoplasm (Suen et al., 2008). The inhibitor, mdivi-1, can effectively downregulate apoptosis by reducing the mitochondrial outer membrane permeabilization, which is directly regulated through attenuation of mitochondrial fission dynamics. This raises the possibility that mdivi-1 represents a novel class of therapeutic agent applicable to many neurodegenerative diseases (Cassidy-Stone et al., 2008). Additionally, apoptotic fission is, at least in part, mediated by the translocation of Drp1 from the cytosol to the mitochondria. The polypeptide, PPD1, can delay apoptosis by blocking Drp1 translocation to the mitochondria, thus preventing their fragmentation and cyt C release (Cereghetti et al., 2010). Recently, Li et al. (2018) have found that inhibition of Drp1 may represent a novel and effective strategy for the treatment of prion diseases. These investigators found that the suppression of Drp1 expression inhibited prion-induced mitochondrial fragmentation and ameliorated PrP^106–126^-induced neurite loss and synaptic abnormalities in primary neurons. Moreover, RNAi targeting Drp1 could also improve the neuronal cell viability and reduce the neuron apoptosis (Li et al., 2018). In the AD, inhibition of excessive Drp1-mediated mitochondrial fission may represent a new therapeutic strategy, since Drp1 inhibition can ameliorate Aβ-mediated mitochondrial dysfunction and synaptic depression in neurons and significantly reduce the Aβ deposition in the brains of AD mice. Moreover, heterozygote Drp1 knockout mice exhibit no effects in terms of mitochondrial and synaptic viability (Manczak et al., 2012; Baek et al., 2017).

Disrupted mitochondria contain oxidized proteins and damaged mitochondrial DNA, factors that are detrimental to cells. To limit the impairment caused by defective mitochondria, cells activate a protective mechanism through which damaged mitochondria are eliminated by mitophagy (Kim et al., 2007). Many studies have revealed a protective role for mitophagy in several deleterious situations, indicating that mitophagy is a good candidate target for therapeutic intervention (Santos et al., 2011). Rapamycin is capable of preventing apoptosis in rotenone-challenged neurons, and this protective effect is due to enhanced mitophagy since the level of mitochondrial colocalization with lysosomes is increased, and the mitochondria could be found within autophagy double-membrane structures during the autophagy process (Pan et al., 2009). Additionally, the PINK1/parkin pathway clearly has a critical role in the degradation of damaged mitochondria by mitophagy (McBride, 2008), with loss-of-function mutations in either PINK1 or parkin leading to an autosomal recessive form of PD, accompanied by the accumulation of dysfunctional mitochondria in neurons as a consequence of impaired mitophagy (Narendra et al., 2008). In *Drosophila*, the overexpression of PINK1 or parkin in PINK1 knockout flies reverses dopaminergic neuron degeneration and mitochondrial dysfunction to levels similar to those observed in parkin mutant flies and can be rescued by the overexpression of parkin (Yang et al., 2006). These findings indicate that interventions that stimulate mitophagy to maintain mitochondrial fidelity could represent a novel approach to delay neurodegenerative processes in PD (Gao et al., 2017).

In conclusion, the machinery regulating mitochondrial dynamics may represent a novel therapeutic target in neurodegenerative diseases, and comprehensive clinical studies are now required to investigate the potential of mitochondrial dynamics as a therapeutic target.

## Author Contributions

TZ wrote the manuscript. QW and WS assisted with the figures. J-LC and BQ critically reviewed the manuscript before final submission. All authors have read and approved the final manuscript.

## Conflict of Interest Statement

The authors declare that the research was conducted in the absence of any commercial or financial relationships that could be construed as a potential conflict of interest.
